# Actin network evolution as a key driver of eukaryotic diversification

**DOI:** 10.1242/jcs.261660

**Published:** 2024-08-09

**Authors:** Katrina B. Velle, Andrew J. M. Swafford, Ethan Garner, Lillian K. Fritz-Laylin

**Affiliations:** ^1^ Department of Biology, University of Massachusetts Dartmouth, Dartmouth, MA 02747, USA; ^2^ Department of Biology, Middlebury College, Middlebury, VT 05753, USA; ^3^ Department of Molecular and Cellular Biology, Harvard University, Cambridge, MA 02138, USA; ^4^ Department of Biology, University of Massachusetts Amherst, Amherst, MA 01003, USA

**Keywords:** Actin, Cytoskeleton, Evolution

## Abstract

Eukaryotic cells have been evolving for billions of years, giving rise to wildly diverse cell forms and functions. Despite their variability, all eukaryotic cells share key hallmarks, including membrane-bound organelles, heavily regulated cytoskeletal networks and complex signaling cascades. Because the actin cytoskeleton interfaces with each of these features, understanding how it evolved and diversified across eukaryotic phyla is essential to understanding the evolution and diversification of eukaryotic cells themselves. Here, we discuss what we know about the origin and diversity of actin networks in terms of their compositions, structures and regulation, and how actin evolution contributes to the diversity of eukaryotic form and function.

## Teaser

**Summary:** Actin polymer networks are essential to eukaryotic cell form and function. This Review highlights what we know about the evolution and diversification of actin networks.

## Introduction

**Fig. 1.**
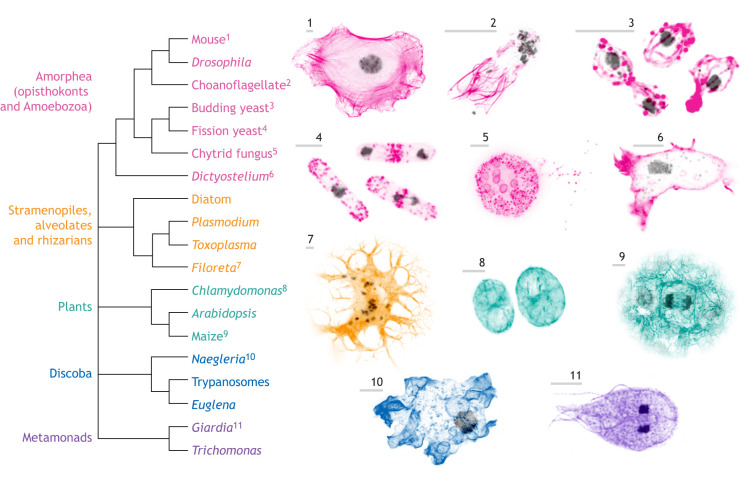
**Diversity of actin cytoskeletal structures across evolution.** The cladogram (left) illustrates the phylogenetic relationships among selected major eukaryotic lineages. Example species are numbered, and the corresponding cytoskeletal structures are shown to the right with actin polymer staining matched to the color coding of the tree, and DNA staining (where available) in gray. Scale bars: 5 μm. This figure was adapted from Velle and Fritz-Laylin (2019), with permission from Elsevier, and is not published under the terms of the CC-BY license of this article. For permission to reuse, please see Velle and Fritz-Laylin (2019). Additional images were provided by Thibaut Brunet (choanoflagellate), Stephanie Brody (chytrid fungus), Clelia Amato (*Dictyostelium*) and Masayuki Onishi (*Chlamydomonas*).

Since sharing a common ancestor well over a billion years ago, eukaryotes have diversified into several major groups, each of which encompasses a wealth of genetic diversity (Fig. 1). Eukaryotic cells can be found in any shape imaginable, from the intricate snowflake-like forms of foraminifera to the needle-like symmetry of diatoms. Eukaryotic cells also vary in size over six orders of magnitude, from ‘picoeukaryotic’ cells that measure less than 1 μm (Courties et al., 1994; Derelle et al., 2006), to neurons that can grow to meters in length. The behaviors of these cells also vary: although some eukaryotic cells are encased in rigid cell walls and obtain energy from the sun, others are shapeshifters that deform themselves to crawl across surfaces and engulf prey. Though there is growing interest in understanding the mechanisms underlying the diversity of eukaryotic cell form and function, we have only a handful of genetically tractable ‘model systems’ – such as mammalian cell lines, *Drosophila*, *Arabidopsis* and yeast – with which to study these mechanisms at the molecular level. Because most eukaryotic model systems come from only two major groups – plants and the ‘yeast-to-human’ opisthokont lineage (Fig. 1) – whether and to what degree their biology is shared with eukaryotes from other lineages, such as the SAR clade (which comprises stramenopiles, alveolates and rhizarians) or Discoba (Fig. 1), remains unknown.

Despite their variability, all eukaryotic cells possess three key features: membrane-bound organelles, heavily regulated cytoskeletal networks and complex signaling cascades. Although it is widely accepted that these features were present in the last eukaryotic common ancestor (Fritz-Laylin et al., 2010; Katz, 2012; Koonin, 2010), how they evolved and diversified amongst eukaryotic lineages is poorly understood and remains a central question for evolutionary and cell biologists. What is clear, however, is that these eukaryotic hallmarks evolved together and remain inextricably intertwined. Because actin and microtubule cytoskeletons are evolutionarily ancient and interface with membranes and signaling cascades to drive a wide variety of eukaryotic phenotypes, understanding the evolution of these cytoskeletal networks is crucial to understanding the evolution of eukaryotic life itself. Here, we focus on how the evolution and diversification of cytoplasmic actin networks and their regulators have shaped the evolution of eukaryotic phenotypes.

## Actin networks interface with other hallmark eukaryotic systems

The intertwining of the actin cytoskeleton with other cell systems allows it to trigger cell-level changes, such as directed cell migration and morphogenesis, in response to molecular signals. These behaviors are driven by the ability of actin monomers to polymerize into filaments that can push and pull on cellular objects. To ensure that actin networks assemble and disassemble at precise times and locations, dozens of actin-binding proteins regulate actin dynamics. These regulators also mediate the intimate relationships between actin and other cell systems, including signaling cascades, membranes and microtubules.

Complex signaling networks are widespread across the eukaryotic tree, and many components of these networks evolved prior to the last eukaryotic common ancestor, including kinases (Grebe and Stock, 1999; Leonard et al., 1998) and second messengers such as cyclic nucleotides (de Mendoza et al., 2014) and Ca^2+^ fluxes (Marchadier et al., 2016). Actin polymer assembly can be triggered by such signaling cascades to prompt swift mechanical responses to intracellular and extracellular signals. For example, white blood cells and amoebae can sense nearby bacteria via chemoreceptors that trigger cascades involving kinases, Ca^2+^ and phospholipids (Artemenko et al., 2014; Consalvo et al., 2019; Futosi et al., 2013). These signaling cascades activate actin polymerization toward the source of the chemical gradient to power rapid movement toward the bacterial prey. Moreover, signaling cascades can alter actin networks and the activity of other cell systems such as microtubules (Guan et al., 2023). The integration of actin with signaling systems therefore allows for holistic cellular responses to internal and external cues.

Most actin networks are assembled at membranes and maintain this connection throughout their lifetimes (Mullins et al., 2018). Animal cells and amoebae, for example, assemble a thick layer of actin at the plasma membrane. This ‘actin cortex’ supports the delicate membrane from within and defines the shape of these cells. Similarly, bundles of parallel actin filaments can push against the plasma membrane to form finger-like filopodial protrusions or microvilli (Blake and Gallop, 2023; Morales et al., 2023). Membrane-associated actin networks can also define cell morphology indirectly, as in the actin-mediated localization of enzymes that expand fungal and plant cell walls (Hernández-González et al., 2018; Schuster et al., 2012; Wightman and Turner, 2008). In addition to controlling cell shape, membrane-associated actin assembly also regulates countless other cell functions, including mitochondrial division (Fung et al., 2023; Hatch et al., 2014), autophagy (Campellone et al., 2023; Monastyrska et al., 2008) and endocytosis (Abouelezz and Almeida-Souza, 2022; Skruzny, 2022), making the tight coupling of actin and membranes essential to the form and function of eukaryotic cells.

The actin cytoskeleton also cooperates with other cytoskeletal polymers, including microtubules. Interactions between actin and microtubules are widespread and include both direct contact and indirect co-regulation (Pimm and Henty-Ridilla, 2021). Recently, actin filaments have been observed to grow along microtubule lattices (Nakos et al., 2022) and have even been discovered inside the lumen of individual microtubules (Paul et al., 2020; Ventura Santos et al., 2023), highlighting the tight connections between these two cytoskeletal systems. Like actin, microtubule polymer dynamics are tightly controlled through many layers of regulation. Whereas actin filaments are flexible and often short, microtubules are stiff and can span the length of the entire cell. Moreover, although actin assembly is typically associated with membranes, microtubules are often polymerized from protein matrices known as microtubule-organizing centers (Wu and Akhmanova, 2017). These differences endow each system with unique properties, and cooperation between actin and microtubules underpins a wide variety of complex cellular functions, including cell division, intracellular transport and cell motility (Pimm and Henty-Ridilla, 2021). During animal cell division, for example, astral microtubules contact membrane-associated actin networks and work together to position the mitotic spindle using the cytoskeletal motors myosin 10 (MYO10) and dynein (Kwon et al., 2015).

In addition to these cytoplasmic roles, there is an increasing appreciation for the function of actin in the nucleus, including in DNA repair (Caridi et al., 2018), replication fork stress (Lamm et al., 2020), chromatin remodeling (Baarlink et al., 2017) and transcriptional regulation (Yoo et al., 2007). Although these functions have been well characterized in very few eukaryotic lineages, they likely also contribute to the evolution of actin networks.

## The many layers of actin regulation

**Fig. 2.**
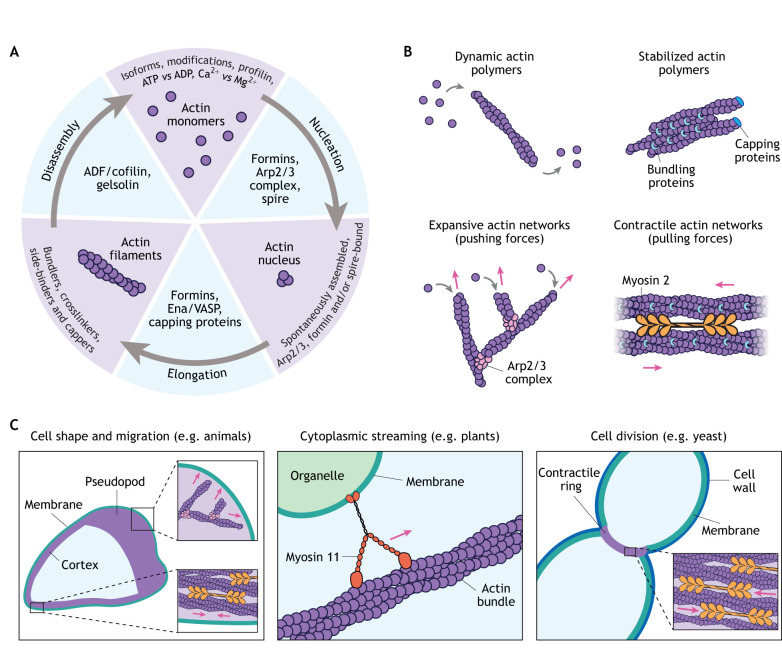
**The many layers of actin regulation.** (A) Actin filaments are regulated at every stage of their lifecycle. Many eukaryotic genomes encode multiple actin ‘isoforms’. The biochemical activity of these distinct actins can further vary by the addition of post-translational modifications (e.g. acetylation), the bound nucleotide (ATP versus ADP) and the bound divalent cation (Ca^2+^ versus Mg^2+^). Actin monomer-binding proteins like profilin can also regulate actin by preventing spontaneous filament assembly. Actin polymerization in cells typically relies on nucleator proteins. Filament length is then regulated by proteins that prevent elongation (e.g. capping proteins) and actin elongation factors (e.g. formins, Ena/VASP). Finally, disassembly of filaments by proteins like ADF/cofilin produces actin monomers that can be recycled into new filaments. (B) Actin is regulated at the level of polymer networks. Actin-binding proteins regulate the dynamics of actin polymers and networks, resulting in distinct network architectures with distinct biophysical properties. For example, Arp2/3-derived networks are branched and useful for pushing on membranes, whereas actomyosin networks are contractile and can produce pulling forces. (C) Multiple actin networks regulate actin-dependent phenotypes. Actin-dependent cell migration often relies on branched actin network growth to push the membrane at the front of the cell and simultaneous contraction of actomyosin networks at the back (left). Intracellular trafficking can be mediated by myosin motor proteins walking along actin filaments or bundles to drag organelles and other objects through the cytoplasm (middle). Fungi, animals and other organisms from the Amorphea group (see Fig. 1) rely on the contraction of actin networks by myosin 2 motors to mediate cytokinesis (right). Myosin 2 and the Arp2/3 complex are depicted as in B.

Understanding the evolution of actin regulation is key to understanding the evolution and diversification of eukaryotic cells. Actin regulation can be divided into three major steps: (1) nucleation, the formation of a stable cluster of actin monomers; (2) elongation, the addition of monomers to the growing end of a filament; and (3) disassembly, the depolymerization of actin filaments, which replenishes the pool of actin monomers within the cell. At each step, the activities of actin-binding proteins give rise to a variety of network architectures and dynamics that mediate diverse cellular phenotypes (Fig. 2).

Actin nucleation does not typically occur spontaneously in cells, despite intracellular monomeric actin concentrations often being orders of magnitude higher than those at which purified actin will spontaneously assemble into filaments (Koestler et al., 2009; Pollard et al., 2000; Wegner and Isenberg, 1983). Although cellular actin concentrations are high, much of the monomeric actin is sequestered by actin monomer-binding proteins (Skruber et al., 2018) – most notably profilins – that prevent spontaneous nucleation (Pollard and Cooper, 1984). The resulting low concentration of free monomeric actin forms a kinetic barrier to spontaneous actin nucleation. To overcome this kinetic barrier, cells use factors called nucleators to initiate actin polymerization only at appropriate times and places (Velle and Fritz-Laylin, 2019). Formin proteins, for example, stabilize actin monomers to form a nucleus (Breitsprecher and Goode, 2013; Gould et al., 2011; Heimsath and Higgs, 2012), whereas the Arp2/3 complex typically uses the actin-related proteins (Arps) Arp2 and Arp3 to template a new actin filament on the side of a pre-existing filament (Mullins et al., 1998). Actin nucleators are, in turn, regulated by dedicated proteins and protein complexes that are activated by any number of upstream signaling pathways. Many of these pathways are stimulated at internal or plasma membranes, resulting in a tight association of actin networks with lipid bilayers. Crawling mammalian cells, for example, rely on small GTPases, kinases and membrane phospholipids to activate ‘nucleation-promoting factors’, such as the SCAR/WAVE complex, which activate the Arp2/3 complex to initiate branched actin network assembly at the plasma membrane (Bieling and Rottner, 2023).

Once nucleated, actin filament elongation can be enhanced by factors that increase the local actin monomer concentration. These factors include formins, which processively associate with the growing end of a filament and recruit profilin-bound actin (Breitsprecher and Goode, 2013). Similarly, elongation factors of the Ena/VASP family and the Arp2/3 activator WASP (also known as WAS in mammals) can bind the growing end of actin filaments along with actin monomers to speed actin assembly (Bieling et al., 2018). Actin elongation can also be negatively regulated by capping proteins, which bind the growing end of the filament to block elongation (Edwards et al., 2014; Isenberg et al., 1980). Variability in actin monomers themselves can add additional layers of complexity to actin filament assembly. For example, binding of ATP rather than ADP (Pollard, 1986) and/or Mg^2+^ rather than Ca^2+^ (Selden et al., 1986) enhances actin polymerization, whereas post-translational modifications like acetylation can alter actin dynamics and regulation (A et al., 2019; Terman and Kashina, 2013). Many species, including humans, also express distinct actin ‘isoforms’ (i.e. actin proteins encoded by separate gene loci, rather than splice variants) that are usually extremely similar in sequence but can have tissue-specific localization (Kashina, 2020; Skruber et al., 2018).

In addition to building new actin networks, cells must depolymerize actin filaments that are no longer useful, a process that also serves to replenish the pool of actin monomers (Goode et al., 2023). Recent work has highlighted that, like actin nucleation, actin disassembly is a highly regulated process, with distinct sets of proteins dedicated to turnover of different actin networks (Goode et al., 2023). Filament disassembly is often mediated by actin depolymerizing factor (ADF)/cofilin proteins. Cofilin binds actin filaments and changes the local stiffness and twist, resulting in breaks between cofilin-bound actin and adjacent subunits (Hocky et al., 2021). In addition to disassembling individual actin filaments, cells must disassemble higher-order actin structures. For example, actin filaments can be tethered together in bundles (Fig. 2B) by proteins like fascins, and these actin bundles can be preferentially depolymerized by specialized disassembly factors (Rajan et al., 2023).

Variation in actin regulation at the molecular, polymer and cellular levels produces a wide variety of network architectures (Fig. 2B) and phenotypes (Fig. 2C). For example, the branched actin networks generated by the Arp2/3 complex are heavily crosslinked and generate pushing forces (Bieling et al., 2016). These networks can drive protrusion of the leading edge of a migrating cell (Wu et al., 2012) and power membrane invagination during endocytosis (Aghamohammadzadeh and Ayscough, 2009; Yarar et al., 2005). Meanwhile, formins build unbranched filaments, which can be organized by myosin 2 motors into contractile actin networks (Fernandez-Gonzalez and Harris, 2023). These actomyosin networks are responsible for the contractility of the actin cortex (Kelkar et al., 2020), retraction of the rear of a migrating cell (Koehl and McNally, 2002) and cytokinesis (Laplante et al., 2015). Myosin activity in the rear of migrating cells can also promote actin filament disassembly by pulling apart actin filaments to create additional ends that can depolymerize (Wilson et al., 2010). Balancing actin assembly, stabilization and disassembly also creates networks with different dynamic properties, from short-lived filaments that push the membrane forward during cell migration (Mueller et al., 2017) to the relatively stable actin bundles in filopodia (Mallavarapu and Mitchison, 1999). Other actin networks serve as tracks for motor proteins that drive cytoplasmic streaming in plants and other organisms (Lu and Gelfand, 2023; Palevitz et al., 1974), as well as cargo delivery to daughter cells in budding yeast (Pruyne et al., 1998) (Fig. 2C). Each layer of actin regulation often uses overlapping subsets of regulators, making the control of actin networks more akin to a series of dials rather than individual switches.

## Complex actin networks evolved prior to eukaryotic diversification

**Fig. 3.**
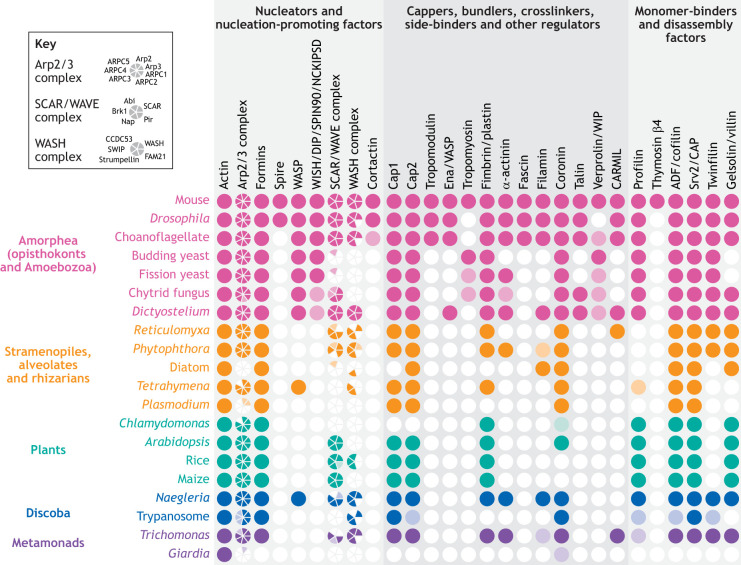
**Core actin regulators are widespread across eukaryotes.** A conservative estimate of actin and actin-binding protein distribution across eukarya shows strong conservation in many protein families. High-confidence hits (darker colors) and low-confidence hits (lighter colors) were identified using a mutual-best-BLAST-hit approach. Because of the conservative nature of this method, ‘absence’ might indicate either actual absence in the group, missing data or significant protein sequence divergence (for example, we did not recover a tropomyosin homolog in *Drosophila*, despite its known presence; Goins and Mullins, 2015). High-confidence hits were identified as reciprocal-best-BLAST hits with a bit score greater than 50 using human, *Drosophila*, budding yeast, *Dictyostelium* and *Arabidopsis* sequences from UniProt proteomes as queries. Low-confidence hits were identified as a top three hit with a bit score greater than 50 using queries based on sequences we previously identified (Prostak et al., 2021).

The core of the actin cytoskeleton, actin itself, is encoded in genomes from all sequenced eukaryotes (Fig. 3) and must therefore have evolved prior to the emergence of the last eukaryotic common ancestor. Actin is the founding member of a diverse family of Arps, whose sequences and function vary within and between species (Frankel and Mooseker, 1996). The best-known Arps are the Arp2 and Arp3 subunits of the Arp2/3 complex, which nucleate branched actin networks. Other family members include Arp1 (also known as centractin), a member of the dynein regulator complex dynactin, as well as Arp4 to Arp9, which have been shown to have chromatin-related functions in opisthokont species (Mullins, 2013). Because actin and Arps are found in species across the eukaryotic tree (Goodson and Hawse, 2002), we can infer that this gene family arose and diversified prior to the last eukaryotic common ancestor.

In addition to numerous actins and Arps, animal cells have dozens of actin network regulators that mediate a wide range of cellular functions. Homologs of many of these actin regulators are also conserved outside the animal lineage, including in fungi, plants and single-celled species. Indeed, simple BLAST searches reveal many key actin regulators in every major eukaryotic lineage (Fig. 3). These ubiquitous actin regulators include actin monomer-binding proteins, nucleators (formin family proteins and the Arp2/3 complex) and their activators, capping proteins, and actin filament-binding proteins. Previous work has also identified myosin motor proteins in species from across the eukaryotic tree (Kollmar and Mühlhausen, 2017). Because of their widespread conservation across eukarya, it is likely that these actin regulators also evolved prior to the last eukaryotic common ancestor. Moreover, we can use the conservation of these key actin regulators to predict actin phenotypes of both extant and extinct eukaryotic species.

The conservation of Arp2/3 and its activators across most eukaryotic lineages (Fig. 3) suggests that branched actin networks were present at the dawn of eukaryotic life. Branched actin networks drive a wide variety of essential cell functions in diverse eukaryotic lineages. Animals and fungi, for example, rely on branched actin networks to push the plasma membrane inward for endocytosis (Aghamohammadzadeh and Ayscough, 2009; Yarar et al., 2005). Many cells also use branched actin networks to push on their plasma membrane to deform themselves and crawl (Fritz-Laylin and Titus, 2023). The best-studied form of cell crawling is that of highly adherent fibroblasts of animals. These cells use force-responsive branched actin networks to push out their fronts (Bieling et al., 2016; Mueller et al., 2017). Related forms of Arp2/3-driven cell migration can also be found in other major eukaryotic lineages, including fungi (Fritz-Laylin et al., 2017), Discoba (Velle and Fritz-Laylin, 2020), metamonads (Kusdian et al., 2013) and amoebae of the amoebozoan lineage (Pollard, 2022).

In addition to genes required for branched actin network assembly, genes encoding proteins that nucleate unbranched actin networks – such as formin family proteins – are also conserved across the eukaryotic tree (Chalkia et al., 2008; Prostak et al., 2021; van Gisbergen et al., 2018). Because formins generate filaments and bundles that can serve as tracks for myosin motors (Johnston et al., 1991; Pruyne et al., 2004), and because motors that drive long-range transport are also widespread across the eukaryotic tree (e.g. members of the myosin 5 and myosin 11 families) (Kollmar and Mühlhausen, 2017), the ability to transport subcellular cargoes using actin filaments appears to be a common hallmark of eukaryotic cells. For example, animals use actin networks for trafficking in neurons (reviewed in Venkatesh et al., 2020), whereas plants use actin networks to drive cytoplasmic streaming – the mixing of cytosol and organelles that is essential for efficient light capture and growth (Tominaga et al., 2013). Species from less familiar lineages, like the apicomplexan parasite *Toxoplasma*, also use actin and myosin for intracellular trafficking (Carmeille et al., 2021).

However, not all actin networks are universal across the eukaryotic tree. Because contractile actin networks require myosin 2, these networks must therefore be restricted to cells that express these motors. Based on current data sets (Kollmar and Mühlhausen, 2017), myosin 2 is phylogenetically limited to a single lineage, the Amorphea (which is the group that includes animals, fungi and Amoebozoa), with one exception. *Naegleria*, an amoeboflagellate from the Discoba lineage, has also been shown to have myosin 2 homologs (Fritz-Laylin et al., 2010). These myosin 2 homologs are thought to have originated through horizontal gene transfer, and their functions remain unknown (Kollmar and Mühlhausen, 2017; Velle and Fritz-Laylin, 2020). Nevertheless, the functions of contractile networks of many species within Amorphea are well defined and include contractile-ring-based cytokinesis (Spudich and Clarke, 1974; reviewed in Brunet, 2023). The restriction of contractile actin networks to species with myosin 2 is consistent with non-contractile-ring-based mechanisms of cytokinesis outside Amorphea (reviewed in Hammarton, 2019), including in plants (Sinclair et al., 2022), *Giardia* (Hardin et al., 2017) and trypanosomes (Sladewski et al., 2023).

Taken together, the deep conservation of many key actin regulators indicates that branched actin networks that provide pushing forces, as well as unbranched actin networks that facilitate intracellular trafficking, are widespread amongst eukaryotic lineages. Moreover, these activities likely evolved prior to the last eukaryotic common ancestor. However, not all modern eukaryotes build identical actin networks. Indeed, differences in actin network structure, dynamics and regulation are key to the diversity of form and function amongst eukaryotic cell types and species.

## Diversification of actin and actin regulators gives rise to phenotypic diversity

**Fig. 4.**
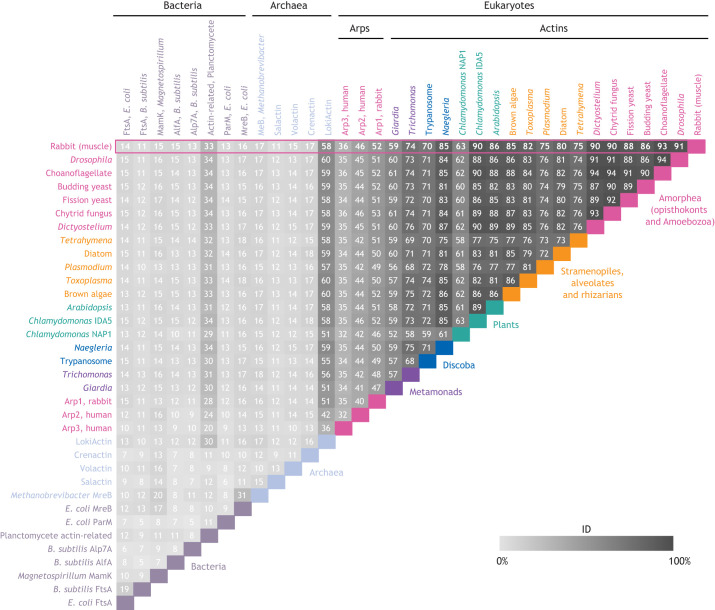
**Actin-like proteins from bacteria and archaea share little sequence identity with eukaryotic actins.** The percentage amino acid sequence identity (ID) for each comparison of actins, Arps or actin-like proteins is shown. All sequences were used to generate a multiple sequence alignment using MUSCLE with defaults in Jalview (Edgar, 2004; Waterhouse et al., 2009). Pairwise comparisons were made covering the length of each full sequence. Sequences from Velle and Fritz-Laylin (2020) were supplemented with the following additional sequences: *Escherichia coli* FtsA (GenBank: AAA23817.1), ParM (GenBank: CAD6159787.1) and MreB (GenBank: QPA16956.1); *Bacillus subtilis* FtsA (UniProt: P28264); *Magnetospirillum magneticum* MamK (UniProt: Q2W8Q6); *B. subtilis* AlfA (UniProt: E9RJG4) and Alp7A (GenBank: ACU27363.1); planctomycete *Uabimicrobium amorphum* actin-related protein (UniProt: A0A5S9IL03); *Methanobrevibacter sp. TLL-48-HuF1* MreB (UniProt: A0A9E7LEF2); *Halobacterium salinarum* Salactin (GenBank: AAG18772.1); *Haloferax volcanii* Volactin (UniProt: D4GU28); *Pyrobaculum califonditis* Crenactin (UniProt: A3MWN5.1); *Lokiarchaeum sp.* (strain GC14_75) LokiActin (UniProt: A0A0F8XG85); human Arp2 (UniProt: P61160) and Arp3 (UniProt: P61158); *Drosophila melanogaster* actin (UniProt: P02574); choanoflagellate *Monosiga brevicollis* actin (UniProt: A9V489); chytrid fungus *Batrachochytrium dendrobatidis* actin (GenBank: KAK5673302.1); and *Arabidopsis thaliana* actin (GenBank: AEE78168.1). See Table S1 for the full alignment.

Although many actin network components are conserved among eukaryotic lineages (Fig. 3), there are many examples where diversification of specific proteins has resulted in diversification of key eukaryotic phenotypes. Even actin itself has diversified within and across lineages (Sehring et al., 2007; Onishi et al., 2016; Avasthi et al., 2024; Stoddard et al., 2017). For example, the actin of the green alga *Chlamydomonas* has undergone duplication and divergence (Kato-Minoura et al., 1998), resulting in one copy that is similar to its ancestral sequence (Fig. 4; IDA5, which shares 90% sequence identity with rabbit muscle actin, ACTA1) and another copy that has diverged enormously (NAP1, which shares 63% sequence identity with rabbit muscle actin). The divergence of NAP1 from other eukaryotic actins makes this protein resistant to actin depolymerization caused by the actin polymerization inhibitor latrunculin B (Onishi et al., 2016), a toxin produced by sea sponges that is thought to serve as a broad-spectrum chemical defense that targets actin (Gillor et al., 2000). Although, as a freshwater alga, *Chlamydomonas* is unlikely to encounter latrunculin B in the wild, actin is a common target of natural toxins, likely because it is highly conserved and essential to all eukaryotes. When treated with latrunculin B, IDA5 filaments disassemble, and *Chlamydomonas* upregulates expression of latrunculin B-resistant NAP1, thereby protecting the cell. Divergence of actin proteins can also occur without gene duplication. The apicomplexan parasite *Toxoplasma*, for example, has a single divergent actin (Dobrowolski et al., 1997) whose polymers undergo rapid turnover *in vitro* (Hvorecny et al., 2023 preprint), a finding that is consistent with the highly dynamic actin networks observed in living *Toxoplasma* cells (Periz et al., 2017).

Core actin regulators have also undergone evolutionary divergence leading to phenotypic diversification. An obvious example is the huge variety of formins in different eukaryotic species. These actin nucleators, which are defined by their FH2 domain that mediates actin assembly, are likely to have been present in the last eukaryotic common ancestor. Since that time, formin family proteins have undergone rampant domain swapping (Chalkia et al., 2008; van Gisbergen et al., 2018), resulting in a huge variety of associated biochemical activities, subcellular localizations and biological functions (Chalkia et al., 2008; Prostak et al., 2021; van Gisbergen et al., 2018). Although much more stable in sequence, the Arp2/3 complex has also undergone diversification in specific lineages (Velle and Fritz-Laylin, 2023). For example, the Arp2 subunit has independently duplicated and diverged within the *Drosophila* lineage. The resulting additional Arp2 proteins have tissue-specific functions in testes, where they build structures important for sperm development (Schroeder et al., 2020; Stromberg et al., 2023). Regulators controlling actin turnover have also diverged in specific lineages. For example, the ADF/cofilin family of actin disassembly factors has undergone expansion within the plant lineage, resulting in two ADF proteins that function in *Arabidopsis* pollen tube development (Wang et al., 2023). Pollen tube growth involves the establishment of a pH gradient from the base to the tip of the growing cell. Each of the two ADF proteins functions best at a different pH, allowing localized activity in different regions of the pollen tube. These are only a few of the many examples of how divergence of individual actin regulators can result in drastic shifts in eukaryotic phenotype.

Another mechanism underlying cytoskeleton-dependent eukaryotic diversification is the loss of actin regulatory genes in specific eukaryotic phyla. This mechanism explains some of the differences between the complex actin cytoskeletons of animal cells and the simple actin networks of yeast. Recently, lineages that diverged from yeast and mushrooms early in fungal evolution have been shown to possess key actin regulators that are missing from yeast, indicating that at least some of the simplicity in yeast actin networks is due to gene loss within the fungal lineage (Prostak et al., 2021). These deeply branching fungi (which include chytrid fungi) also have similar phenotypes to animal cells, including the ability to crawl using branched actin networks, suggesting that the yeast lineage lost the ability to crawl concurrently with the loss of specific actin regulators (Fritz-Laylin et al., 2017; Prostak et al., 2021). Another clear example of loss of specific actin regulators coinciding with the absence of actin-dependent phenotypes is the cytoskeleton of *Entamoeba.* This amoebozoan lacks a number of actin regulators required for branched actin-dependent crawling motility. Consistent with this reduced complement of actin regulators, *Entamoeba* has been shown to lack branched actin-dependent crawling and instead crawls using myosin 2 contractility (Maugis et al., 2010).

Some eukaryotic species have even more limited complements of actin regulators. Perhaps the most extreme example of this is the gut parasite *Giardia*, from the metamonad lineage. *Giardia* has a divergent actin but no other detectable homologs of known actin regulatory proteins (Paredez et al., 2011). Whether *Giardia* lacks actin-binding proteins because its lineage never had them or because of secondary gene loss is not fully understood. Supporting the idea of secondary loss, however, is the presence of key actin regulators in the genome of *Trichomonas* (Kusdian et al., 2013), another parasite that groups with *Giardia* in evolutionary trees. In any event, the divergence of *Giardia* actin proteins from other eukaryotic actins, combined with an absence of regulators, suggests that actin may be free to diversify in the absence of constraints imposed by actin-binding proteins, as has been proposed for microtubule network evolution (Kennard et al., 2024 preprint).

## Actin-like polymers are widespread in bacteria and archaea

The broad conservation of actin and Arps across eukaryotic lineages suggests that this protein family pre-dates eukaryotic diversification. Accordingly, multiple proteins that share structural similarity to eukaryotic actins have also been discovered in bacteria and archaea (Charles-Orszag et al., 2024). These proteins contain the actin family superfold and form filaments in an ATP- or GTP-dependent manner (Stoddard et al., 2017; Wagstaff and Löwe, 2018). Eukaryotic actin and the actin-like proteins of bacteria and archaea are thought to have evolved from a common polymerizing ancestor within the actin superfold family that includes Hsp70 and DnaK, as well as glucokinase and hexokinase families (Stoddard et al., 2017). Interestingly, this is not the only time polymerization evolved from this family – Glk1, a glucokinase in *Saccharomyces cerevisiae*, independently evolved the ability to polymerize to regulate its enzymatic activity (Stoddard et al., 2020).

**Fig. 5.**
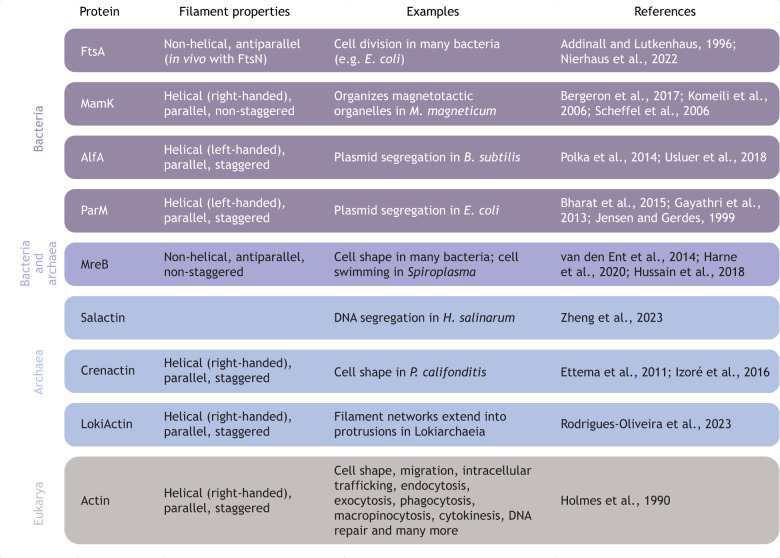
**Bacterial and archaeal actin-like proteins have variable filament structures and each drives limited phenotypes.** The figure displays examples of actins and actin-like proteins present in bacteria, archaea and eukarya. Some actin-like proteins are present in both archaea and eukarya. Most actin-like protein functions relate to cell shape or division (Addinall and Lutkenhaus, 1996; Bergeron et al., 2017; Bharat et al., 2015; Ettema et al., 2011; Gayathri et al., 2013; Harne et al., 2020; Holmes et al., 1990; Hussain et al., 2018; Izoré et al., 2016; Jensen and Gerdes, 1999; Komeili et al., 2006; Nierhaus et al., 2022; Polka et al., 2014; Rodrigues-Oliveira et al., 2023; Scheffel et al., 2006; Usluer et al., 2018; van den Ent et al., 2014; Zheng et al., 2023).

Despite their structural and biochemical similarities, the sequence diversity of bacterial and archeal actin-like proteins is greater than that of eukaryotic actins and Arps; most share less than 16% sequence identity with rabbit muscle actin and share similarly low levels of sequence identity with each other (Fig. 4; Table S1). One exception to this divergence can be found in a planctomycete bacterium that engulfs other bacteria and whose genome encodes an actin-like protein that is 34% identical to rabbit muscle actin (Shiratori et al., 2019). A second exception are the actin-like proteins found within Asgard archaea – an archeal group widely believed to be the closest living relative to eukaryotes (Akıl et al., 2022; Rodrigues-Oliveira et al., 2023; Spang et al., 2015; Survery et al., 2021; Zaremba-Niedzwiedzka et al., 2017). For example, LokiActin from Lokiarchaeia shares 58% sequence identity with rabbit muscle actin and forms two-stranded filaments with the same right-handed helicity as eukaryotic actins (Fig. 5) (Rodrigues-Oliveira et al., 2023). Meanwhile, many other actin-like proteins from bacteria and archaea form a wide variety of filament structures (Fig. 5), including both right-handed (Bergeron et al., 2017; Izoré et al., 2016) and left-handed helical filaments (Bharat et al., 2015; Gayathri et al., 2013; Usluer et al., 2018), flat membrane-binding filaments formed from anti-parallel protofilaments (Nierhaus et al., 2022; van den Ent et al., 2014), three-stranded filaments (Bergeron and Kollman, 2023 preprint), and even highly organized fifteen-stranded structures (Koh et al., 2019). These divergent actin-like proteins display a wide range of filament dynamics, from the extremely stable filaments of MreB (van Teeffelen et al., 2011) to those of ParM, Salactin and Alp7A, which show dynamic instability (Drew and Pogliano, 2011; Garner et al., 2004; Zheng et al., 2023), and FtsA filaments, which treadmill with the tubulin homolog FtsZ (Bisson-Filho et al., 2017).

Like eukaryotic actin and Arps, bacterial and archaeal actin-like proteins can interact with other factors to regulate filament dynamics. These bacterial regulators include sequestering proteins such as AimB (Werner et al., 2020), the proposed MreB nucleator RodZ (Bratton et al., 2018; Dion et al., 2019), and DNA-bound proteins that can nucleate filaments and/or bind to their ends and stabilize them (Polka et al., 2014). Filaments of Crenactin (an actin-like protein in the crenarchaean *Pyrobaculum calidifontis*) can be depolymerized by Arcadin-2 – a mechanism that appears similar to that of eukaryotic actin and ADF/cofilin proteins (Izoré et al., 2016). Also similar to eukaryotic actin regulation, Asgard archaean genomes have been found to encode putative homologs of profilins and gelsolin (Akıl and Robinson, 2018; Akıl et al., 2020). Impressively, profilins from Lokiarchaeia and Heimdallarchaea can bind rabbit muscle actin and prevent spontaneous nucleation (Akıl and Robinson, 2018; Survery et al., 2021). Although it remains possible that understudied bacterial lineages include species with larger numbers of actin-binding proteins that await discovery, the handful of known actin-binding proteins outside eukaryotes is no match for the bounty of actin bundlers, crosslinkers, nucleators, elongators and severing proteins that characterize the eukaryotic actin cytoskeleton.

As may be expected from the limited set of regulators found in any given species, the networks formed by individual bacterial and archaeal actin-like proteins are less diverse than those formed by eukaryotic actins (Charles-Orszag et al., 2024). Moreover, these polymers are often specialized for a single cellular function, such as segregating DNA, creating a rod shape or aiding in organizing cell division (Fig. 5). Despite these limitations, bacterial and archaeal actin-like proteins share key characteristics with eukaryotic actins and Arps, including the ability to bind membranes and organelles (as has been observed for MreB and MamK; Salje et al., 2011; Komeili et al., 2006), interact with tubulin homologs (as demonstrated by the interaction of FtsA with FtsZ; Pichoff and Lutkenhaus, 2007) and be regulated by Ca^2+^ (Akıl et al., 2022). These similar traits might reflect core requirements of polymer systems that span length scales, integrate information and mediate cell-level responses.

## Conclusion

The eukaryotic actin cytoskeleton is regulated at the molecular, polymer and cellular levels to produce networks with variable dynamics and architectures to drive diverse phenotypes. From the expansive branched actin networks that drive cell migration in animal cells to the stable actin bundles that support polarized growth of fungi, the versatility of actin networks underscores the critical role of actin in shaping eukaryotic cell form and function. As we trace the evolutionary roots of actin, we find its vestiges in bacteria and archaea. Although the basic architecture of actin-like proteins is conserved, the limited diversity of regulators and network architectures in bacteria and archaea underscores the evolutionary leap that occurred within eukaryotes. Moreover, the paucity of known regulators of actin-like proteins in bacterial and archaeal species, along with the striking actin sequence diversity within these lineages, suggests a model for actin evolution that is similar to what has been proposed for microtubule evolution (Kennard et al., 2024 preprint). Under this model, the evolution of cytoskeletal polymers is constrained and guided by their interactions with polymer-binding proteins (Fig. 2). This model predicts that polymers in lineages with many binding proteins undergo less diversification than those in lineages with fewer binding proteins. Given the lack of observable actin-binding proteins in *Giardia*, this model could also account for the relative divergence of its actin from those of other eukaryotes. Moreover, this model implies that the evolution of the actin cytoskeleton can only be understood in the context of its regulatory networks.

Despite being more conserved than bacterial and archaeal actin, eukaryotic actin networks exhibit significant variability within specific lineages, leading to distinct cellular phenotypes. From divergent actin sequences in organisms like *Chlamydomonas* to the loss of actin regulators in yeast, the diversification of the actin cytoskeleton underpins distinct eukaryotic phenotypes. Moreover, because the actin cytoskeleton is interconnected with other fundamental cellular systems, its evolution and diversification are pivotal to shaping the eukaryotic cellular landscape.

To understand how actin network evolution drives eukaryotic diversification, we must first define the structure and regulation of actin networks in diverse species – particularly those outside the opisthokont lineage. Because historically neglected organisms are likely to have both conserved and previously undiscovered actin phenotypes, determining the mechanisms governing their regulation will require tool development to allow both forward and reverse genetics and/or biochemical analysis of actin regulation. This work will not only shed light on new actin phenotypes but will likely help us understand actin regulation in well-studied lineages. For example, historical work on actin phenotypes of species within the amoebozoan lineage was instrumental in laying the foundation for our understanding of actin biology in animals (reviewed in Pollard, 2022). More recently, our own work on the actin phenotypes of chytrid fungi has helped us understand the regulation of white blood cell motility (Fritz-Laylin et al., 2017). Moving forward, we eagerly await the application of these approaches to other lineages whose species are equally important for life on Earth.

## Supplementary Material

10.1242/joces.261660_sup1 Supplementary information

Table S1. Full alignment of protein sequences shown in Fig. 4.
