# Supplementary material for: Actin network evolution as a key driver of eukaryotic diversification
Source: J Cell Sci. 2024 Aug 9;137(15):jcs261660. doi: 10.1242/jcs.261660 (PMC12050087; doi:10.1242/jcs.261660)
Supplement: Supplementary information [file joces-137-261660-s1.pdf]

Table S1. Full alignment of protein sequences shown in Fig. 4.

Available for download at
https://journals.biologists.com/jcs/article-lookup/doi/10.1242/jcs.261660#supplementary-data
